# Decreased miRNA expression in Klinefelter syndrome

**DOI:** 10.1038/s41598-017-16892-3

**Published:** 2017-11-30

**Authors:** Laura Cimino, Michele Salemi, Rossella Cannarella, Rosita A. Condorelli, Giorgio Giurato, Giovanna Marchese, Sandro La Vignera, Aldo E. Calogero

**Affiliations:** 1 Department of Clinical and Experimental Medicine, University of Catania, Catania, 95123 Italy; 2 Oasi Institute for Research on Mental Retardation and Brain Aging (IRCCS), Troina, 94018 Italy; 3 Laboratory of Molecular Medicine and Genomics, Department of Medicine, Surgery and Dentistry “Schola Medica Salernitana”, University of Salerno, Baronissi, 84081 Italy; 4 Genomix4Life Srl, Department of Medicine, Surgery and Dentistry “Schola Medica Salernitana”, University of Salerno, Baronissi (SA), 84081 Italy

## Abstract

The widelyvariable phenotypic spectrum and the different severity of symptoms in men with Klinefelter syndrome (KS) suggest a role for epigenetic mediators. Therefore, the aim of this study is to evaluate the possible involvement of miRNAs in the clinical manifestations of KS. To accomplish this, we performed a transcriptome analysis in peripheral blood mononuclear cells (PBMCs) of 10 non-mosaic KS patients, 10 aged-matched healthy men and 10 aged-matched healthy female controls with normal karyotype. After RNA extraction from PBMC and the preparation of RNA libraries, the samples were sequenced using next generation high-throughput sequencing technology. Expression profiling analysis revealed a significant differential expression of 2 miRNAs in KS compared to male controls. In particular, MIR3648 resulted significantly (q-value < 0.0001) down-regulated by −19.084- fold, while MIR3687was strongly down-regulated (q-value < 0.0001) considering KS patients. These results were confirmed by qRT-PCR. The functional analysis of the two transcripts showed that they seem to play a role in breast cancer, hemopoietic abnormalities, immune defects and adipocyte differentiation and fat cell maturation. Therefore, we speculate that both miRNAs may play a role in the immune and metabolic disorders and in the risk of breast cancer development in men with KS.

## Introduction

Klinefelter syndrome (KS) is a common sex-chromosome aneuploidy with an estimated prevalence of 1 inevery 660 male births^1,2^. However, most of the men with KS remain undiagnosed or are being diagnosed later in life^3^. KS is characterized by a 47,XXY karyotype in about 80–90% of men, whereas the remaining cases are given by chromosome mosaicis.369*m (e.g. 47,XXY/46,XY), additional sex chromosomes (e.g. 48,XXXY; 48,XXYY; 49,XXXXY) or X chromosome structural abnormalities (e.g. 47,X,iXq,Y)^4^. The lowtestosterone levels observed in a high proportion of these men could explain the classical characteristics of KS, such as tall stature, gynecomastia, and infertility^5^, as well as some of other disorders that have an increased prevalence in KS, including osteoporosis^6^, metabolic syndrome, and type 2 diabetes mellitus^7^. The other disorders associated with KS, such as an increased incidence of mediastinictumors, neurocognitive and psychiatric disturbances, are less readily explained by hypotestosteronemia^5,8^. The wide phenotypic spectrum and the different severity of symptoms in non-mosaic KS patients suggest a role for epigenetic factors.

In recent years, a number of studies have investigated gene expression profiles of KS to improve the evaluation of the molecular basis of KS phenotype. Nevertheless, the mechanisms leading to germ cell degeneration and consequently azoospermia are still unclear. It has been hypothesized that an altered gene dosage, escaping inactivation on the supernumerary X-chromosome, might affect the development or cause degeneration of germ cells^9^. However, data on the role of X-linked genes on testicular function are inconsistent^10^. Therefore, evaluation of molecular regulators of gene expression, such as microRNA (miRNAs), could help to understand the molecular background in KS men. Nevertheless, so far,only few studies have explored the role (if any) of miRNAsin KS men.

miRNAs are non-coding small RNAs (about 22 nucleotides) that play a role in the post-transcriptional regulation of gene expressionand protein translation^11,12^. Abnormal miRNA expression has been reported to be involved in the occurrence and development of various diseases, such as cancer, cardiovascular disease, mental retardation, fetal growth restriction^13–18^. Growing evidence has shown that miRNAs play critical roles in regulating male germ cell development and are essential for epigenetic regulation of mitosis, meiosis and spermiogenesis^19–21^.

On this basis, the present study was undertaken to evaluate the expression of miRNAs in men with KS by high-throughput sequencing technology and to assess the possible involvement of miRNAs in the pathogenesis and clinical manifestations of KS. miRNA expression was compared to that of healthy age-matched men and women with normal karyotype.

## Results

We performed a transcriptome analysis on peripheral blood mononuclear cells (PBMCs) of 10 non-mosaic KS patients and 10 male controls. Expression profiling analysis revealed the significant differential expression of 73 transcripts in KS men compared to controls, highlighting two clusters of differentially expressed genes composed by 60 down- and 13 up-regulated transcripts in KS.

**Figure 1:**
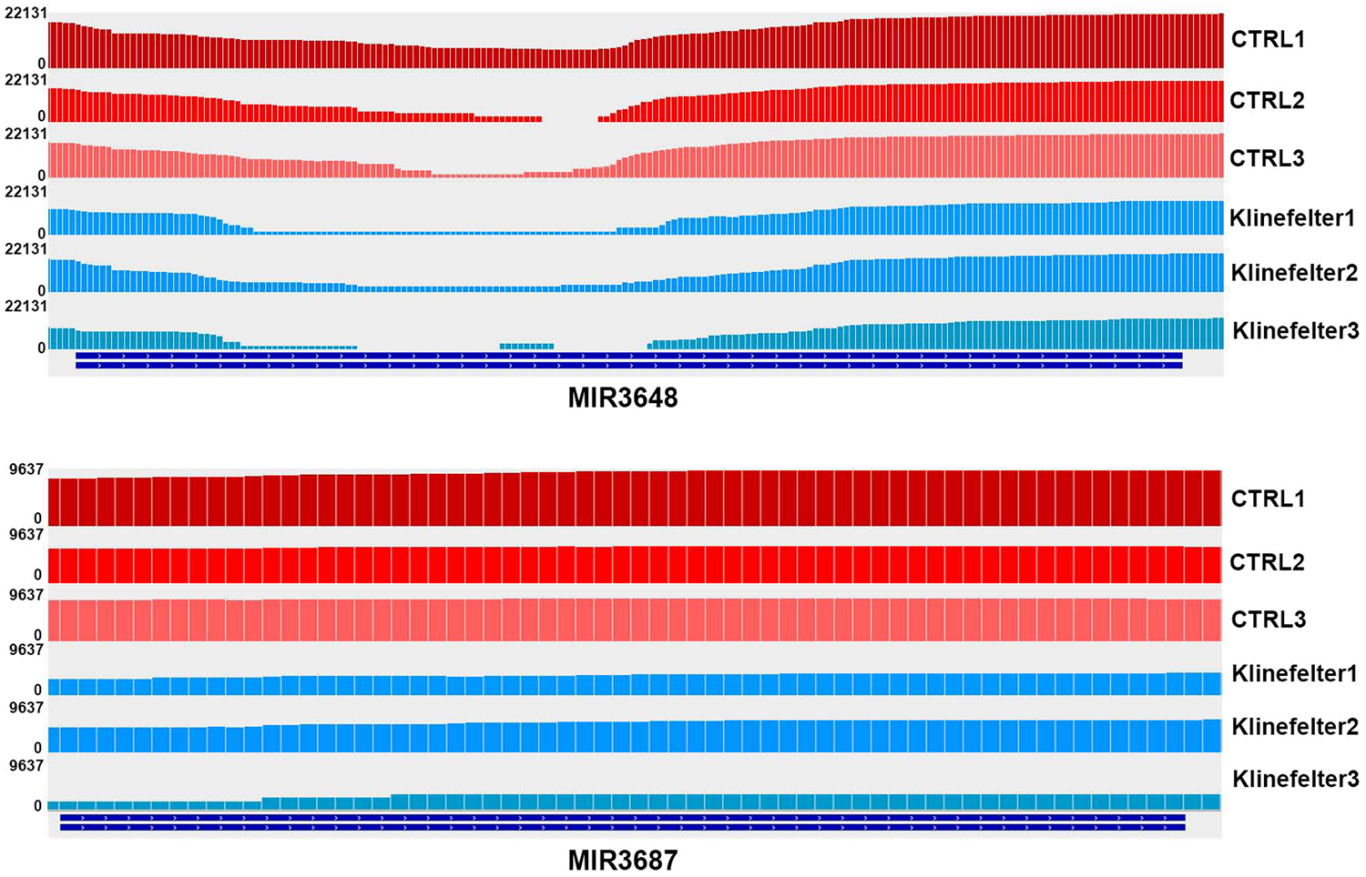
Screen-shot of MIR3648 and MIR3687. Screen-shot from IGV - Integrative Genomics Viewer for MIR3648 and MIR3687. Three control samples and three Klinefelter samples among the ten samples are displayed. The numbers along the side of each sample represent the maximum reads coverage in that specific region.

Among the down-expressed genes, we found a significant differential expression of two miRNA precursors in KS compared to controls (Table 1). MIR3648 resulted significantly (q-value < 0.0001) down regulated by −19.084-fold, whereas MIR3687 was strongly down regulated (q-value < 0.0001) (Fig. 1).

**Table 1:** miRNAs differentially expressed in men with KS men compared to normal age-matched controls.

Gene	Locus	Fold-Change	p value	q value
miRNA-3687	21:9826202–9826263	DOWN_regulated	0.00005	0.017811
miRNA-3648	21:9825831–9826011	−19,084	0.00005	0.017811

**Figure 2:**
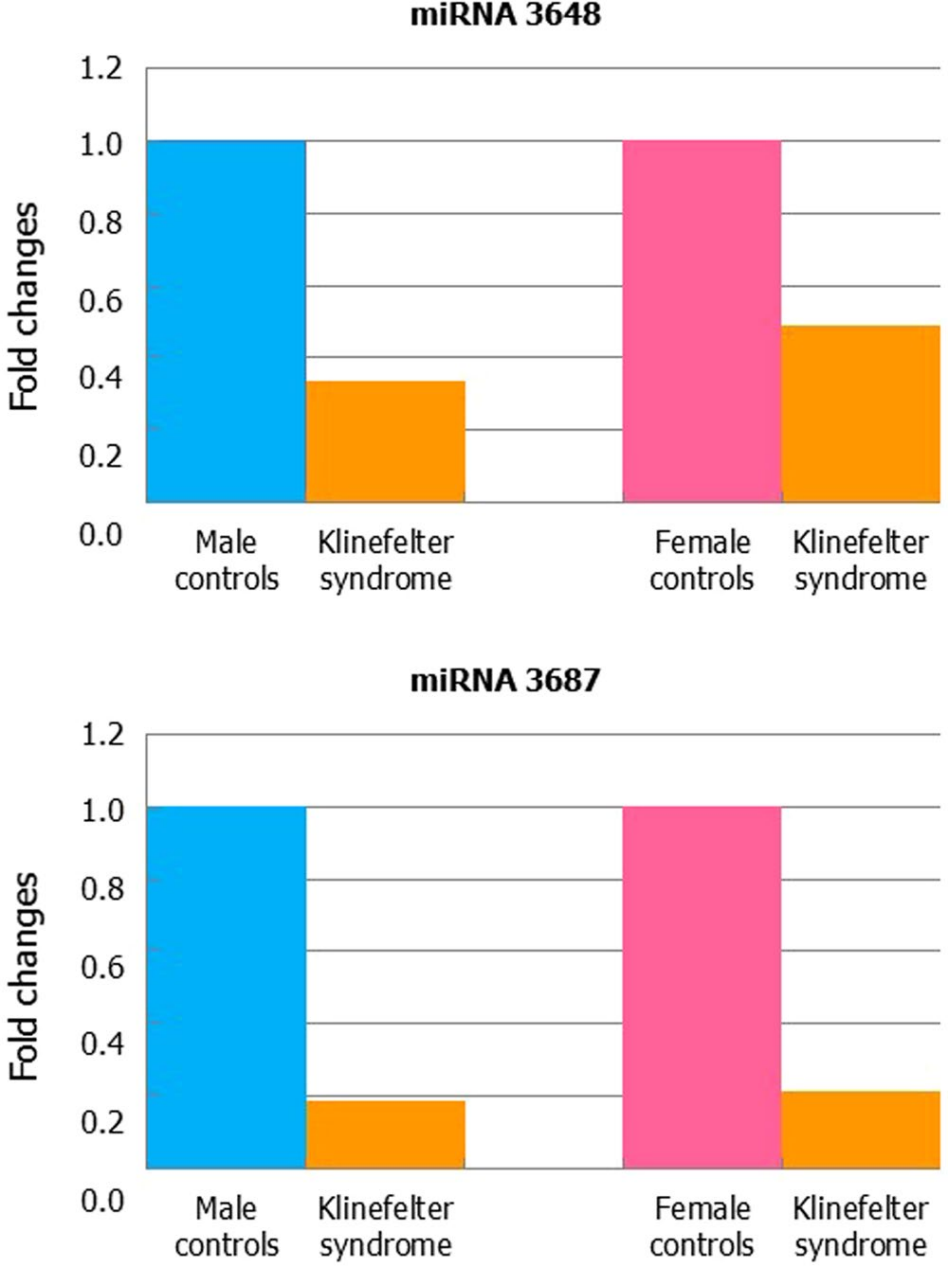
MIR3648 (upper panels) and MIR3687 (lower panels) qRT-PCR expression in leukocytes of men with Klinefelter syndrome compared to healthy male or female controls.

The results obtained by NGS analysis were confirmed by qRT-PCR. Indeed, miRNA 3648 and miRNA 3687 were significantly under expressed in KS compared to both male (Fig. 2, upper panels) and female (Fig. 2, lower panels) controls.

## Discussion

KS is characterized by varying degrees of cognitive, social, behavioural, learning difficulties and in adulthood also by primary testicular failure with small testes, hypergonadotropic hypogonadism, tall stature, and eunuchoid body proportions. The phenotype is variable ranging from “near-normal” to a significantly affected individual suggesting the involvement of epigenetic mediators in their presentation. The aim of the present study is,indeed, to evaluate the possible involvement of miRNAs in KS clinical manifestations.

To gain insight into the character of miRNAs in men with KS, we employed high-throughput sequencing technology to globally study miRNA expression profiles in KS patients compared to age-matched controls. Further sequence tag analysis revealed that two miRNAs were aberrantly expressed: MIR3648 had decreased expression and MIR3687 was not expressed in PBMCs from KS men compared to controls. Not all the other miRNAs showed differential expression between KS and normal controls, suggesting a rather stable miRNAs expression profile in KS men. Thus so far, knowledge on miRNAs in KS is limited. Both the two deregulated miRNAsmap on chromosome 21.

Men with KS have an increased risk of developing breast cancer and several studies have addressed this issue. The mechanism is not well understood. Several publications have looked at the miRNA expression profiles in breast cancer cell lines and tumor tissues. A closer inspection of the patterns of deep sequencing reads mapped to the miR-3648 suggests that it may not be a *bona fide*miRNA, since it does not show a read pattern compatible with small RNA processing^22^. The role that miR-3648 may play in cancer is yet unknown. miR-3648 was described in solid tumors^23^, and it may be processed differently to suppress the expression of different target genes^22^. This opens up the possibility that miR-3648 exerts different functions in different kinds of cancer, or exerting different functions in the same tumor tissue thus causing, or contributing to tumor heterogeneity and plasticity^24^. Recently, Emmadi and colleagues found that miR-3648 positively correlates with estrogen receptor (ER)-positive breast carcinoma^25^. The expression characteristic of miRNA during the development of Down syndrome (DS) fetuses showed that miR-3648 and miR-3687 were up regulated more than 50% in the DS fetal cord blood mononuclear cells. This suggests that the differentially expressed miRNAs may be involved in the hemopoietic abnormalities and the immune defects of DS fetus^26^.

Adults with KS have five-fold increased risk for developing metabolic syndrome compared to age-matched controls^6^. Insulin resistance and metabolic syndrome are present in 24% and 7%, respectively, of 89 KS children as young as 4–12 years of age^27^. Both studies showed that KS patients had truncal obesity. miRNA have been suggested as therapeutic targets in metabolic syndrome due to their role in the maturation of fat cells and the differentiation of adipocytes from mesenchymal stromal stem cells. Recently, the role of metformin to suppress the differentiation of human preadipocytes was evaluated. After 2 weeks of 5 mM metformin treatment, 27 miRNAs were significantly upregulated and 6 miRNAs were down regulated in human visceral preadipocytes (HPrAD-vis) compared to cells control. In particular, the expression of miR-1246 and miR-3687 increased following metformin administration, while the expression of miR-378 family members decreased. This finding suggest that adipocyte differentiation in regulated by miRNAs. This result identifies miR-1246, miR-3687 and miR-422a as promising therapeutic targets for the treatment of visceral obesity^28^.

To date, several genes have been identified as targets of miR-3687, including PPARGC1B (PPARG coactivator 1β), MEMO1 (mediator of cell motility 1) and GDF7. PPARGC1B encodes a protein that stimulates the activity of several transcription factors and nuclear receptors, including ERα, nuclear respiratory factor 1 and glucocorticoid receptor. The encoded protein may be involved in fat oxidation, non-oxidative glucose metabolism and the regulation of energy expenditure. This protein is downregulated in prediabetic and type 2 diabetes mellitus patients^29^. Certain allelic variations in this gene increase the risk of development of obesity.

MEMO 1 is a copper-dependent redox protein with an essential role in migration and metastasis. It is involved in breast carcinogenesis via regulating insulin-like growth factor-I receptor-dependent signalling events^30^.

GDF7 encodes a secreted ligand of the transforming growth factor-β proteins superfamily. It is required for normal seminal vesicle development, branching morphogenesis and cell differentiation. Mice lacking a functional copy of this gene exhibit absence of some spinal dopaminergic neurons and brain defects, male sterility, and premature death^31^.

## Conclusions

In conclusion, NGS analysis showed that non-mosaic KS men have a lower expression of MIR-3648 and MIR-3687 compared to normal controls. The functional analysis of the deregulated transcripts suggests that these miRNAs may play a role in the molecular mechanisms involved in germ cells degeneration, neurological disorders and the risk of development of cancer and obesity that are more likely to occur in KS patients. Results are to be considered preliminary because gene expression was measured in blood cells. Further validating and functional studies are needed to confirm these findings and to better understand the molecular basis of the variable phenotype found in KS patients.

## Material and Methods

### Selection criteria for patients and controls

This study was approved by the Ethics Committee of University teaching Hospital of “Policlinico-Vittorio Emanuele, University of Catania, (Catania, Italy), trial registration number 49/2015/PO (Register of the Ethics Committee opinions). All methods were performed in accordance with the relevant guidelines and regulations. All participants were asked for and provided their informed consent. We performed a transcriptome analysis on PBMCs of 10 non-mosaic KS patients and 10 aged-matched healthy controls. All KS patients (mean age 32.5 ± 3.0 years) had 47,XXY karyotype as shown by cytogenetic investigation conducted on at least 50 metaphase and five out of ten were treated with testosterone enanthate. Normozoospermic controls (mean age 32.0 ± 3.0 years) had a negative history for genetic disease, normal testicular volume and normal reproductive hormones (FSH, LH, total testosterone) levels. Ten healthy age-matched women (mean age 32.0 ± 2.9 years) were also recruited as female controls. All KS and male and female controls were Italians (Eastern Sicily) (Table 2).

**Table 2:** Clinical features and biochemical data of men with non-mosaic Klinefelter syndrome and age-matched controls.

ID	Age (years)	BMI (Kg/m^2^)	LH (mUI/ml)	FSH (mUI/ml)	T (ng/ml)	RTV (ml)	LTV (ml)	TRT
CTL1	34	26.2	7.78	3.5	6.36	15	15	NO
KS1	35	28	24.3	34.9	3.2	1	1	YES
CTL2	32	27.1	4.57	3.69	6.13	22	25	NO
KS2	32	39.9	9.9	19	2.3	2	1.5	NO
CTL3	31	27.3	4.93	4.5	6	18	18	NO
KS3	33	20.1	31.1	55	3.3	1	1	YES
CTL4	31	25.1	2.1	3.5	4.7	16	16	NO
KS4	29	27.3	15	25	2.1	1.5	1.5	NO
CTL5	36	23.6	2.1	3.5	4.7	16	16	NO
KS5	38	22	26.5	33.3	5.17	1.8	1.8	YES
CTL6	24	23	7.78	3.5	6.36	20	20	NO
KS6	23	17.6	11.6	13.7	9.03	5	5	YES
CTL7	24	20	4	3.2	10.5	20	15	NO
KS7	21	29.3	22.2	25.6	1.03	2	1.5	NO
CTL8	50	24.7	5.2	2.08	4.7	21	19	NO
KS8	48	30.1	18	23	5	2	1.4	YES
CTL9	28	24.5	7.04	3.84	5.13	22	21	NO
KS9	26	24.9	29.1	64.6	2.8	2	1.5	NO
CTL10	41	29.6	4.6	4	4.3	15	15	NO
KS10	39	22.5	17.2	35.6	2.6	2	2	NO

ID: identification number;BMI: body mass index;LH: Luteinizing hormone; FSH: follicle stimulating hormone; T: testosterone;RTV: right testicular volume; LTV: left testicular volume; TRT: testosterone replacement therapy; CTL: control; KS: men with Klinefelter syndrome.Normal range: LH: 1.14–8.75 mUI/ml; FSH: 0.95–11.95 mUI/ml; T: 2.5–9.8 ng/ml.

### RNA sequencing

Heparinized venous blood sample was withdrawn from each KS and male and female control. Peripheral blood mononuclear cells (PBMCs) were obtained using Ficoll-Paque (Ficoll Plaque PLUS – GE Healthcare Life Sciences, Piscataway, USA). Total RNA was extracted using the standard RNA extraction method with TRIzol (Invitrogen, Carlsbad, CA, USA). Before use, RNA concentration in each sample was assayed with a ND-1000 spectrophotometer (NanoDrop) and its quality assessed with the Agilent 2100 Bioanalyzer with Agilent RNA 6000 nano kit (Agilent Technologies, Santa Clara, CA, USA).

Indexed libraries were prepared from 1 µg/ea purified RNA with TruSeq Stranded Total RNA (Illumina) Library Prep Kit according to the manufacturer’s instructions. Libraries were quantified using the Agilent 2100 Bioanalyzer (Agilent Technologies) and pooled such that each index-tagged sample was present in equimolar amounts, with final concentration of the pooled samples of 2bnM. The pooled samples underwent cluster generation and sequencing using an Illumina HiSeq. 2500 System (Illumina) in a 2 × 100 paired-end (RNA-seq) format.

### Validation

To validate the results obtained by Next-Generation Sequencing (NGS) analysis, we compared qRT-PCR in 10 KS patients and 10 normal subjects. In this second step, the subjects studied in the NGS analysis were included. Once again, KS cases and controls were recruited after family and/or personal informed consent. RNA extraction from leucocytes of peripheral blood was performed using RNeasy Mini Handbook (Qiagen Sciences, Germantown, USA), following the manufacturer’s protocol. Retro-transcription of 650 ng of total RNA from each sample was then performed in a final volume of 30 μl and the cDNA generated was used as a template for real-time quantitative PCR analysis with gene expression products. For each sample qRT-PCR reactions were carried out in duplicate using 4 μl of cDNA and QuantiTect Probe PCR Master Mix Kit (QIAGEN Sciences, Germantown, PA, USA) in a total volume of 25 μl. QRT-PCR experiments were performed using the Light Cycler 480 (Roche Diagnostics; Mannheim, Germany). The target MIR3648, MIR3587 and the reference gene glyceraldehyde-3-phosphate dehydrogenase (GAPDH) assays were obtained from Applied Biosystems (Carlsbad, CA, USA).

The thermal cycling conditions consisted of one cycle for 2 min at 50 °C, 1 cycle of 15 min at 95 °C and 42 cycles for 15 s at 94 °C followed by 1 min at 60 °C. The amplified transcripts were quantified using the comparative CT method and relative quantification analysis data were played using the comparative ΔΔCt method. The MIR expression level was normalized to GAPDH level and Target Mean Cp definition was used to indicate the mean normalized cycle threshold.

In our case–control study with qRT-PCR we have used all cases and controls and specifically we obtained a mean FC (Fig. 2). The mean was obtained with the Software Version 1.5 supplied with the LightCycler 480. We can conclude that the results confirm the data obtained by the NGS analysis and differences in values reflect the diversity of methods.

### Data analysis

The raw sequence files generated (.fastq files) underwent quality control analysis using FastQC (http://www.bioinformatics.babraham.ac.uk/projects/fastqc/) and the quality checked reads were trimmed with cutadapt^32^ v.1.10 and then aligned to the human genome (hg19 assembly) using STAR v.2.5.2^33^, with standard parameters. Differentially expressed mRNAs were identified using DESeq. 2 v.1.12^34^. Firstly, gene annotation was obtained for all known genes in the human genome, as provided by Ensembl (GRCh37). Using the reads mapped to the genome, we calculated the number of reads mapping to each transcript with HTSeq-count v.0.6.1^35^. These raw read counts were then used as input to DESeq. 2 for calculation of normalized signal for each transcript in the samples, and differential expression was reported as fold change along with associated adjusted p-values (computed according to Benjamini-Hochberg). Differential expression data were further confirmed using Cuffdiff^36^. Raw data are available in ArrayExpress database repository (https://www.ebi.ac.uk/arrayexpress/) with accession number E-MTAB-6107.
